# Editorial: The Upcoming Complications of COVID-19 on Recovered Patients: Molecular Mechanisms and Therapeutic Opportunities

**DOI:** 10.3389/fmolb.2022.922541

**Published:** 2022-05-23

**Authors:** Rana Jahanban-Esfahlan, Seyed Hassan Seradj, Zohreh Amoozgar, William C. Cho

**Affiliations:** ^1^ Department of Medical Biotechnology, Faculty of Advanced Medical Sciences, Tabriz University of Medical Sciences, Tabriz, Iran; ^2^ Department of Medicinal Chemistry, School of Pharmacy, Shiraz University of Medical Sciences, Shiraz, Iran; ^3^ Edwin L. Steele Laboratories, Department of Radiation Oncology, Massachusetts General Hospital and Harvard Medical School, Boston, MA, United States; ^4^ Department of Clinical Oncology, Queen Elizabeth Hospital, Kowloon, Hong Kong SAR, China

**Keywords:** COVID-19, molecular mechanisms, long-term effects, recovered patients, therapeutic opportunities

It has been more than 2 years since the world was struck by the COVID-19 pandemic and its emerging variants as Delta, Omicron, and mixed variants. Although global vaccination efforts are effective in controlling COVID-19, recovered patients experienced numerous short- and long-term complications of COVID-19.

Our Research Topic aims to identify the underlying molecular mechanisms and pathways involved of the COVID-19 pathogenesis in the development/reactivation of underlying diseases/disorders. This probably get some more insights for the development of therapeutics, prediction and prevention of the occurrence of life-long morbidities in recovered patients.

In our Research Topic, two studies focused on the pathogenesis, molecular mechanisms, and therapeutic targets of COVID-19. Rahbar et al. highlighted the role of host serine protease 2 (TMPRSS2) in viral infection. Given endosomal and non-endosomal entry of virus in the host cell. In the non-endosomal pathway, the virus entry through S protein is facilitated through its cleavage by furin, which is further activated by TMPRSS2. Clinical trial results showed Bromhexine hydrochloride as a promising intervention for the treatment of early COVID-19 infection by inhibiting the activity of these enzymes. Transcriptional inhibition of TMPRSS2 has no side effects on healthy organs or normal development and homeostasis in the host.

According to the WHO reports, for COVID-19, the current variants of concern (include B.1.1.7 (Alpha), B.1.351 (Beta), P.1 (Gamma), B.1.617.2 (Delta), and B.1.1.529 (Omicron) arise due to a high rate of the genetic recombination of S1-RBD/S2 mutations/deletions in the spike protein that have an impact on virus activity. In this respect, Hosseini et al. reviewed the origin and evolution, structure, genetic diversity, route of transmission, pathogenesis, new diagnostic and treatment strategies, as well as the psychological and economic impacts of the COVID-19 pandemic on individuals and their lives around the world.

Other two papers focused on the diagnostic tools/biomarkers for COVID-19 detection. Such that, genome editing targets for site-specific insertions, the CRISPR-Cas system was selected as the 2015 Breakthrough of the Year by Science (Doudna and Charpentier, 2014) and the pioneers won the Nobel Prize of 2020 in Chemistry (Westermann et al., 2021). Shademan et al. reviewed the application of CRISPR technology in the detection and treatment of SARS-CoV-2 infection. For example, a CRISPR/Cas9-mediated lateral flow nucleic acid assay has been developed to identify infection using the CRISPR/Cas system (Wang et al., 2020), enabling low-cost point-of-care detection methods to identify SARS-CoV-2 infection in the clinical setting (Azhar et al., 2014). Due to the excellent sensitivity, specificity, and reliability of RNA-guided nucleic acid detection, CRISPR/Cas nuclease has recently shown great potential for developing next-generation molecular diagnostics. However, there are challenges in transferring CRISPR/Cas9 into virus-infected cells, the possibility of off-target activity and mutant viruses should also be addressed. Given the impact of cardiovascular disease in SARS-COV-2 infection and the severity of symptoms, cardiac tropin, a known biomarker of cardiovascular disease, has prognostic value in cardiac diseases and COVID-19. Rasmi et al. evaluated the diagnostic value, pathophysiological mechanisms, and novel assessment methods of troponin, including a novel biosensor for troponin in patients with COVID-19.

The rest of the papers in our Research Topic discussed COVID-19 complications such as respiratory, metabolic, oral, cancer, autoimmune disease, and other factors (age and gender). The lung is the primary organ that is affected by the coronavirus and three papers were related to the respiratory complications of COVID-19. As in a study by Matusali et al., autopsies of confirmed COVID-19 patients showed positivity in spindle-like cells infiltrating the sub-mesothelial stroma and the staining confirmed the mesothelial origin. This finding suggests that SARS-COV-2 disrupts the epithelium and after invading the sub mesothelia promotes pleural fibrosis. This disruption is facilitated by neuropilin-1 (NRP1) expression, a co-receptor of vascular endothelial growth factor (VEGF) with a profibrotic activity. SARS-COV-2 infected cells produce massive levels of cytokines with pro-inflammatory abilities (IFNs) and anti-inflammatory, e.g., interleukin 10 (IL-10) at the same time. Among these cytokines, IL-10 creates a double-edged sword as it can dampen the immune system and simultaneously enhance the production of interferon gamma (IFNγ). The cytokine regulation elevates metalloproteases (MMPs) that, in turn, orchestrate fibrosis in the lungs, a significant cause of mortality observed in COVID-19 patients. Calabrese et al. assessed the pulmonary function and exercise capacity 3 months after recovery from pneumonia in patients with COVID-19. They found that COVID-19 patients with a positive clinical history of pulmonary embolism (PE) had more impaired lung function tests compared to COVID-19 patients with a negative clinical history of PE, including a higher percentage of patients who experienced dyspnoeic with exercise and showed a peripheral capillary oxygen saturation (SpO_2_)< 90% on the 6-min walk test. Importantly, as severe cases of COVID-19 require hospitalizations, and the significant long-term effect is the reduction in gas transfer, in a meta-analysis Guo et al. stressed that the routine respiratory follow-up of COVID-19 patients is necessary.

Given that lung is not the only organ disturbed during COVID-19 infection, Kaviani et al. summarized the pathogenesis of COVID-19 in the heart, kidney, and liver with a focus on metabolic disease. Moreover, Zhou et al. studied the oral complications (such as ageusia and macroglossia) in patients following COVID-19 infection. Finally, the practical strategies for preventing oral complications are summarized, and a rehabilitation plan for patients with oral complications is constructed. In long run, COVID-19 may result in the development of cancers due to the integration of the virus and the host genome. This possibility may arise due to alteration in the immune system and induction of immunoregulatory pathways that are crucial in the surveillance and eradication of deformed host cells, as reviewed by Rahimmanesh et al.

Hosseini et al. discussed the causes and consequences of the multisystem inflammatory syndrome and autoimmune diseases following SARS-CoV-2 infection which may manifest as Guillain-Barré syndrome and systemic lupus erythematosus to provide a clear view of health care providers and researchers. Finally, age and gender significantly influence the COVID-19 outcomes. Here, Hachim et al. have shown differentially expressed genes in lung tissue of male versus female patients and correlated their findings with signaling pathways such as nuclear factor-kappa B as a regulator of inflammation.

Overall, this Research Topic would serve as a resource to expound the underlying molecular mechanisms of long-term complications in COVID-19 recovered patients and pave the way to consider strategies to reduce these morbidities.
